# A Comparison Between Two Definitions of Contrast-Associated Acute Kidney Injury for Long-Term Mortality in Elderly and Non-elderly Patients After Elective Percutaneous Coronary Intervention

**DOI:** 10.3389/fcvm.2021.720857

**Published:** 2021-09-27

**Authors:** Haoming He, Zhebin You, Xueqin Lin, Chen He, Sicheng Zhang, Manqing Luo, Maoqing Lin, Liwei Zhang, Kaiyang Lin, Yansong Guo

**Affiliations:** ^1^Department of Cardiology, Shengli Clinical Medical College of Fujian Medical University, Fujian Provincial Hospital, Fuzhou, China; ^2^Fujian Provincial Key Laboratory of Cardiovascular Disease, Fujian Cardiovascular Institute, Fujian Provincial Center for Geriatrics, Fujian Clinical Medical Research Center for Cardiovascular Diseases, Fuzhou, China; ^3^Fujian Heart Failure Center Alliance, Fuzhou, China; ^4^Department of Geriatric Medicine, Shengli Clinical Medical College of Fujian Medical University, Fujian Provincial Hospital, Fujian Provincial Center for Geriatrics, Fuzhou, China

**Keywords:** different definitions, contrast-associated acute kidney injury, mortality, elderly, percutaneous coronary intervention

## Abstract

**Background:** Contrast-associated acute kidney injury (CA-AKI) is responsible for a substantial proportion of the observed mortality that occurs after percutaneous coronary intervention (PCI), particularly in elderly patients. However, there has been significant and debate over whether the optimal definition of CA-AKI persists over prolonged periods due to variations in the prevalence and effects on prognosis. In this study, we aimed to identify whether different definitions of CA-AKI exert differential impacts on long-term mortality when compared between elderly and non-elderly patients receiving elective PCI.

**Methods:** We prospectively investigated 5,587 consenting patients undergoing elective PCI between January 2012 and December 2018. We considered two classical definitions of CA-AKI from the European Society of Urogenital Radiology (ESUR) and the Acute Kidney Injury Network (AKIN). Multivariable Cox regression analysis was used to investigate the association between CA-AKI and long-term mortality. We also performed interaction and stratified analyses according to age (≤75 or >75 years).

**Results:** The incidence of CA-AKI according to the ESUR and AKIN definitions was 18.7 and 6.1%, respectively. After a median follow-up of 2.1 years, multivariable Cox regression analysis indicated that CA-AKI according to the AKIN definition was a risk factor for long-term mortality in the overall population [hazard ratio (HR) = 2.20; 95% confidential interval (CI): 1.51–3.22; *p* < 0.001]; however, this was not the case for the ESUR definition (HR = 1.27; 95% CI: 0.92–1.76; *p* = 0.153). Further interaction analysis identified a significant interaction between age and the ESUR definition (*p* = 0.040). Stratified analyses also found an association between the ESUR definition and long-term mortality in patients >75 years of age (*p* = 0.011), but not in patients ≤75 years of age (*p* = 0.657).

**Conclusion:** As a stringent definition of CA-AKI, the AKIN definition was significantly associated with long-term mortality in both non-elderly and elderly patients. However, in elderly patients, the more lenient definition provided by the ESUR was also significantly correlated with long-term mortality, which could sensitively identify high-risk elderly patients and may provide a better alternative.

## Introduction

Contrast-associated acute kidney injury (CA-AKI) is a common but deleterious complication of percutaneous coronary intervention (PCI) that increases the duration of hospitalization, healthcare costs, the need for dialysis, and the risk of mortality (1–5). However, the definition used to describe CA-AKI has been the source of significant debate. Owing to the lack of consensus, multiple definitions are used for CA-AKI; these have different thresholds and lead to clear variation in the incidence and prognosis of CA-AKI (6–12). The optimal definition for CA-AKI remains controversial and has yet to be elucidated.

Compared with non-elderly patients, elderly patients undergoing PCI are known to have a higher risk of developing CA-AKI and a poorer prognosis (13–16). Therefore, it is vital that we investigate CA-AKI in the elderly to facilitate prompt prophylactic strategies and improve their prognosis. It is particularly important to identify an appropriate definition of CA-AKI for use in the elderly population. At present, the most widely used definitions for CA-AKI in patients who have undergone PCI are the two classical definitions that were established by the European Society of Urogenital Radiology (ESUR) (17) and the Acute Kidney Injury Network (AKIN) (18). However, it is unclear whether these different definitions of CA-AKI exert differential impacts on long-term mortality when compared between elderly and non-elderly patients.

Therefore, the primary aim of the present study was to investigate the prognostic impact of CA-AKI according to the two classical definitions on long-term mortality among elderly and non-elderly patients receiving elective PCI.

## Materials and Methods

### Study Design and Participants

This was a prospective and observational study that included consenting patients who had been diagnosed with acute coronary syndrome (ACS) and stable coronary artery disease (CAD) and undergoing elective PCI between January 2012 and December 2018 at Fujian Provincial Hospital. The exclusion criteria were as follows: (1) patients with end-stage renal disease [estimated glomerular filtration rate (eGFR) <15 ml/min/1.73 m^2^]; (2) patients who required long-term dialysis; (3) patients who had been exposed to contrast medium for 48 h before or 72 h after the procedure; (4) patients for which pre-procedural or post-procedural serum creatinine (SCr) data were missing or unavailable; (5) patients who took nephrotoxic drugs during hospitalization; and (6) patients who died within 24 h of admission. In total, 5,587 patients were included in the final analysis. Ethical approval was obtained from the Fujian Provincial Hospital Ethics Committee, and the protocol was carried out in accordance with the Declaration of Helsinki (Ethical approval number: K2019-07-011).

### Protocol

SCr concentration was detected at admission and then continuously for at least 3 days after the application of contrast media. Other laboratory indicators, such as routine blood parameters and fasting lipid profile, were also measured on the day of admission or the following morning. PCI procedures were conducted by experienced interventional cardiologists. All patients who underwent elective PCI were administered with low-osmotic and non-ionic contrast media (370 mgI/ml of Iopamiron or 370 mgI/ml of Ultravist). The hydration protocol included an intravenous infusion of 0.9% saline at a rate of 1 ml/kg/h for 12 h during the perioperative period (or 0.5 ml/kg/h in patients with overt heart failure). The strategy used for pharmaceutical therapy was determined by cardiologists in accordance with appropriate guidelines.

### Definitions and Follow-Up

The primary endpoint was all-cause mortality during follow-up. According to the ESUR definition, CA-AKI is defined as an increase in SCr ≥0.5 mg/dl or ≥25% within 72 h after the administration of contrast media (17). According to the AKIN definition, CA-AKI was defined as an increase in SCr ≥0.3 mg/dl or ≥50% within 48 h (18). eGFR was calculated as follows: 186.3 × (SCr)^−1.154^ × (age)^−0.203^ × 0.742 (if the patient was female) in accordance with the Modification of Diet in Renal Disease equation (19). The electronic medical records database was used to collate all clinical, laboratory, and angiographic data. Follow-up data were collated by trained research nurses at outpatient clinical visits or by telephone interviews following hospital discharge.

### Sample Size Estimation

The sample size required for Cox regression analysis was estimated using Power Analysis and Sample Size (PASS) Software (version 15, NCSS, LLC., Kaysville, UT, USA; ncss.com/software/pass). According to a previous study (20), we assumed that CA-AKI (according to the ESUR definition) was associated with a 1.39 hazard ratio (HR) for long-term mortality (probability of event: 0.086). The incidence and variability (standard deviation) of CA-AKI were 0.220 and 0.414, respectively. The power was set to 80%, and the alpha value was set to 0.05. Considering a 10% dropout rate, the required minimum sample size was calculated to be 5,467 patients. Our final sample size (5,587) was therefore appropriate.

### Statistical Analysis

All data were processed and analyzed using the R statistical language (R Foundation for Statistical Computing, Vienna, Austria; version 4.0.2). Continuous variables were analyzed by Student's *t*-test and presented in the form of mean ± standard deviation (SD). Categorical variables were compared using the chi-squared test or Fisher's exact test and summarized as counts (percentage). Cox regression analysis was used to evaluate the prognostic impact of the two different CA-AKI definitions on mortality; results are presented in the form of forest plots. The variables that demonstrated significance in the univariate analysis (*p* < 0.05), or considered to be clinically important, were then used in multivariate regression analysis. We performed formal tests to study the interaction between CA-AKI definition and age (stratified above/below the age of 75 years). We also conducted stratified analyses by age (≤75 or >75 years) to investigate the association between CA-AKI according to different definitions and mortality. The Kaplan–Meier curves were plotted and compared by the log-rank test. A *p*-value < 0.05 (two-sided) was considered to be statistically significant.

## Results

### Patients Characteristics

**Figure 1:**
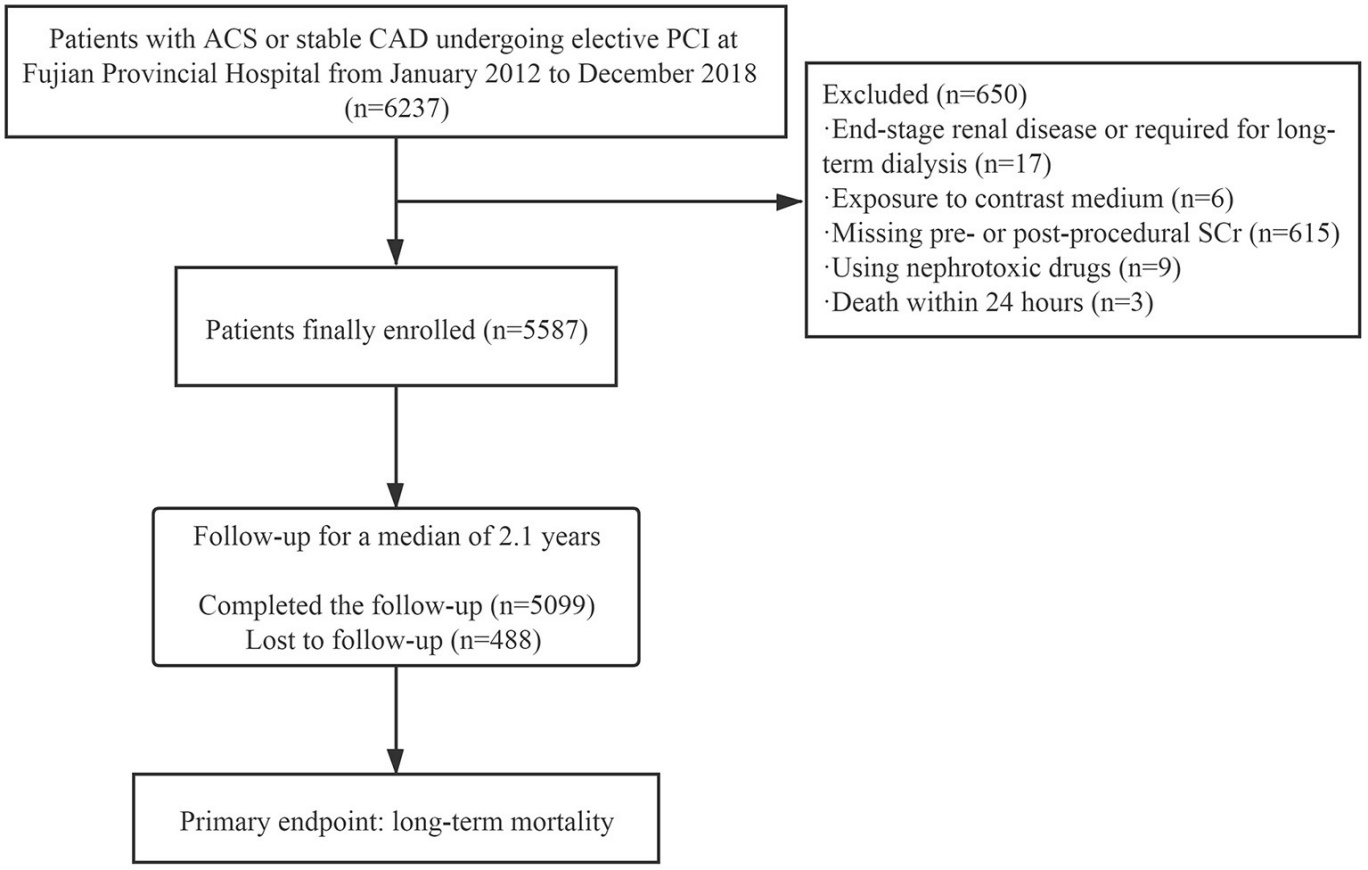
Flowchart showing the procedure used for patient recruitment. ACS, acute coronary syndrome; CAD, coronary artery disease; PCI, percutaneous coronary intervention; SCr, serum creatinine.

Figure 1 shows a flowchart describing the procedure used for recruiting patients. A total of 5,587 cases were recruited; baseline characteristics are summarized in Table 1. The overall mean age was 65.3 ± 10.4 years, and 962 (17.2%) patients were older than 75 years. In total, 1,220 (21.8%) patients were female, and almost one-third of the patients had diabetes mellitus. A small proportion of patients (13.4%) had experienced a previous myocardial infarction (MI), and 83.1% had multivessel disease. Analysis showed that 3.4% of patients presented with a baseline SCr >1.5 mg/dl. The baseline SCr in elderly patients (>75 years of age) was significantly higher than that in non-elderly patients (≤75 years of age) (1.00 vs. 0.90 mg/dl, *p* < 0.001) (Supplementary Figure 1).

**Table 1:** Comparison of baseline characteristics in the overall population and in those with CA-AKI according to both AKIN and ESUR definitions.

**Variables**	**Overall population**	**CA-AKI according to AKIN definition**			**CA-AKI according to ESUR definition**		
	**(*n* = 5,587)**	**No (*n* = 5,245)**	**Yes (*n* = 342)**	***p*-value**	**No (*n* = 4,543)**	**Yes (*n* = 1,044)**	***p*-value**
**Demographics**							
Age, years	65.3 ± 10.4	65.2 ± 10.3	68.0 ± 11.5	<0.001	65.3 ± 10.3	65.8 ± 10.6	0.169
Age > 75 years, *n* (%)	962 (17.2)	868 (16.5)	94 (27.5)	<0.001	757 (16.7)	205 (19.6)	0.025
Sex, female, *n* (%)	1,220 (21.8)	1,119 (21.3)	101 (29.5)	<0.001	876 (19.3)	344 (33.0)	<0.001
**Medical history**							
Previous MI, *n* (%)	746 (13.4)	706 (13.5)	40 (11.7)	0.395	627 (13.8)	119 (11.4)	0.044
Smoker, *n* (%)	2,349 (48.1)	2,227 (48.7)	122 (39.2)	0.001	1,978 (50.0)	371 (40.1)	<0.001
Diabetes, *n* (%)	2,002 (35.8)	1,851 (35.3)	151 (44.2)	0.001	1,599 (35.2)	403 (38.6)	0.042
Hypertension, *n* (%)	3,798 (68.0)	3,541 (67.5)	257 (75.1)	0.004	3,082 (67.8)	716 (68.6)	0.670
Acute MI, *n* (%)	1,697 (30.4)	1,500 (28.6)	197 (57.6)	<0.001	1,259 (27.7)	438 (42.0)	<0.001
Type of CAD				<0.001			<0.001
STEMI, *n* (%)	663 (11.9)	569 (10.8)	94 (27.5)		488 (10.7)	175 (16.8)	
NSTEMI, *n* (%)	1,034 (18.5)	931 (17.8)	103 (30.1)		771 (17.0)	263 (25.2)	
UA, *n* (%)	3,318 (59.4)	3,190 (60.8)	128 (37.4)		2,797 (61.6)	521 (49.9)	
Stable CAD, *n* (%)	572 (10.2)	555 (10.6)	17 (5.0)		487 (10.7)	85 (8.1)	
**Procedure performed**							
Multivessel disease, *n* (%)	4,489 (83.1)	4,206 (83.0)	283 (85.5)	0.263	3,661 (83.3)	828 (82.4)	0.526
LM	472 (8.8)	439 (8.7)	33 (10.0)	0.469	388 (8.8)	84 (8.4)	0.680
LAD	4,840 (89.6)	4,540 (89.5)	300 (90.6)	0.592	3,933 (89.5)	907 (90.2)	0.500
LCX	3,761 (69.6)	3,508 (69.2)	253 (76.4)	0.007	3,062 (69.7)	699 (69.6)	0.980
RCA	3,798 (70.3)	3,551 (70.0)	247 (74.6)	0.088	3,092 (70.3)	706 (70.2)	0.987
Number of stents	1.66 ± 0.87	1.65 ± 0.87	1.68 ± 0.93	0.582	1.67 ± 0.87	1.61 ± 0.87	0.045
Stent length, mm	44.97 ± 26.79	44.88 ± 26.73	46.38 ± 27.75	0.340	45.31 ± 26.85	43.50 ± 26.48	0.052
Contrast volume >150 ml, *n* (%)	3,842 (71.7)	3,602 (71.6)	240 (73.6)	0.467	3,125 (71.6)	717 (72.1)	0.813
**Medical therapy during hospitalization**							
Antiplatelet, *n* (%)	5,578 (99.8)	5,238 (99.9)	340 (99.4)	0.101	4,536 (99.8)	1,042 (99.8)	0.678
Statin, *n* (%)	5,538 (99.1)	5,199 (99.1)	339 (99.1)	1.000	4,497 (99.0)	1,041 (99.7)	0.037
β-Blocker, *n* (%)	4,625 (82.8)	4,330 (82.6)	295 (86.3)	0.092	3,726 (82.0)	899 (86.1)	0.002
CCB, *n* (%)	1,998 (35.8)	1,862 (35.5)	136 (39.8)	0.124	1,639 (36.1)	359 (34.4)	0.321
**Laboratory measurements**							
Serum creatinine, μmol/L	81.32 ± 24.74	81.09 ± 23.69	84.98 ± 37.16	0.057	84.20 ± 24.19	68.83 ± 23.18	<0.001
eGFR, ml/min/1.73 m^2^	89.59 ± 24.04	89.53 ± 23.00	90.51 ± 36.51	0.625	85.88 ± 21.19	105.76 ± 28.64	<0.001
SCr > 1.5 mg/dl, *n* (%)	190 (3.4)	161 (3.1)	29 (8.5)	<0.001	168 (3.7)	22 (2.1)	0.014
WBC, 10^9^/L	7.34 ± 2.27	7.29 ± 2.21	8.12 ± 2.95	<0.001	7.29 ± 2.22	7.55 ± 2.48	0.002
Hemoglobin, g/L	136.97 ± 16.35	137.43 ± 16.04	130.01 ± 19.22	<0.001	137.69 ± 16.17	133.86 ± 16.74	<0.001
PLT, 10^9^/L	222.24 ± 69.02	222.51 ± 69.36	218.14 ± 63.42	0.227	222.11 ± 69.44	222.83 ± 67.18	0.759
Cholesterol, mmol/L	4.23 ± 1.17	4.23 ± 1.17	4.28 ± 1.15	0.371	4.21 ± 1.17	4.30 ± 1.15	0.032
Triglyceride, mmol/L	1.69 ± 1.21	1.68 ± 1.22	1.73 ± 1.16	0.499	1.69 ± 1.25	1.64 ± 1.03	0.163
Low-density lipoprotein, mmol/L	2.68 ± 1.05	2.67 ± 1.05	2.73 ± 1.03	0.289	2.66 ± 1.05	2.75 ± 1.04	0.012

*CA-AKI, contrast-associated acute kidney injury; AKIN, Acute Kidney Injury Network; ESUR, European Society of Urogenital Radiology; MI, myocardial infarction; CAD, coronary artery disease; STEMI, ST-segment elevation myocardial infarction; NSTEMI, non-ST-segment elevation myocardial infarction; UA, unstable angina; LM, left main stem; LAD, left anterior descendant; LCX, left circumflex artery; RCA, right coronary artery; CCB, calcium channel blockers; eGFR, estimated glomerular filtration rate; SCr, serum creatinine; WBC, white blood cells; PLT, platelet*.

### The Incidence of Contrast-Associated Acute Kidney Injury

**Figure 2:**
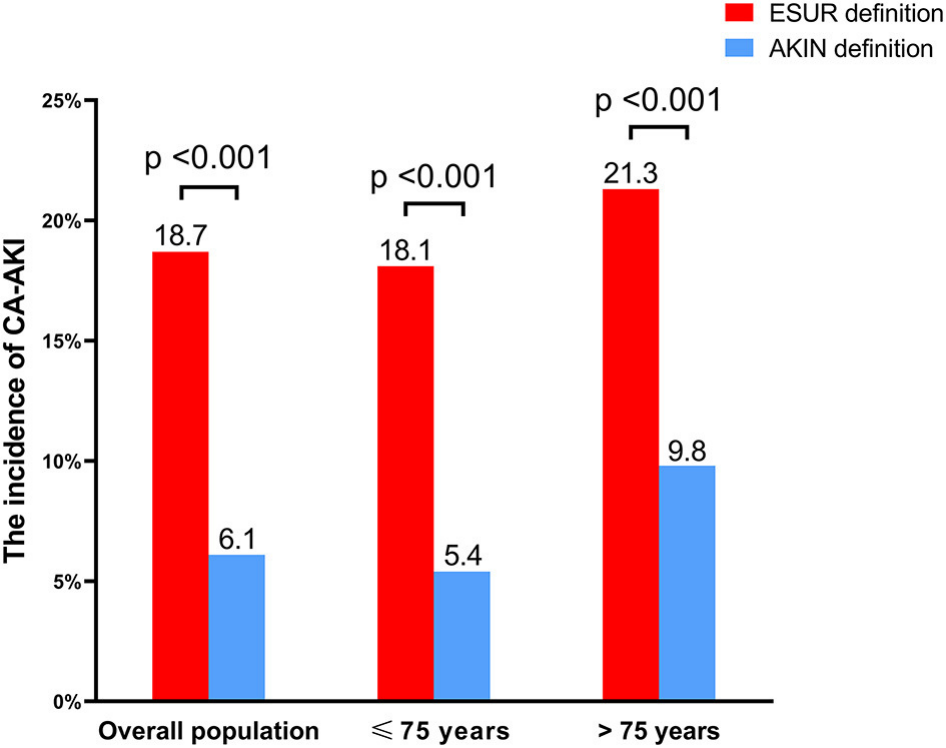
The incidence of CA-AKI according to both AKIN and ESUR definitions. CA-AKI, contrast-associated acute kidney injury; AKIN, Acute Kidney Injury Network; ESUR, European Society of Urogenital Radiology.

In the overall population, 18.7% (*n* = 1,044) of patients were diagnosed with CA-AKI when applying the ESUR definition, compared with only 6.1% (*n* = 342) when applying the AKIN definition. When analyses were stratified by age, the proportion of patients with CA-AKI, as determined by the ESUR definition, was 21.3% in the >75-year-old subgroup, compared with 18.1% in the ≤75-year-old subgroup. Similarly, CA-AKI was detected in 9.8 and 5.4% of patients in these two subgroups when the AKIN definition was applied (Figure 2).

### The Predictive Value of Different Contrast-Associated Acute Kidney Injury Definitions for Long-Term Mortality

**Figure 3:**
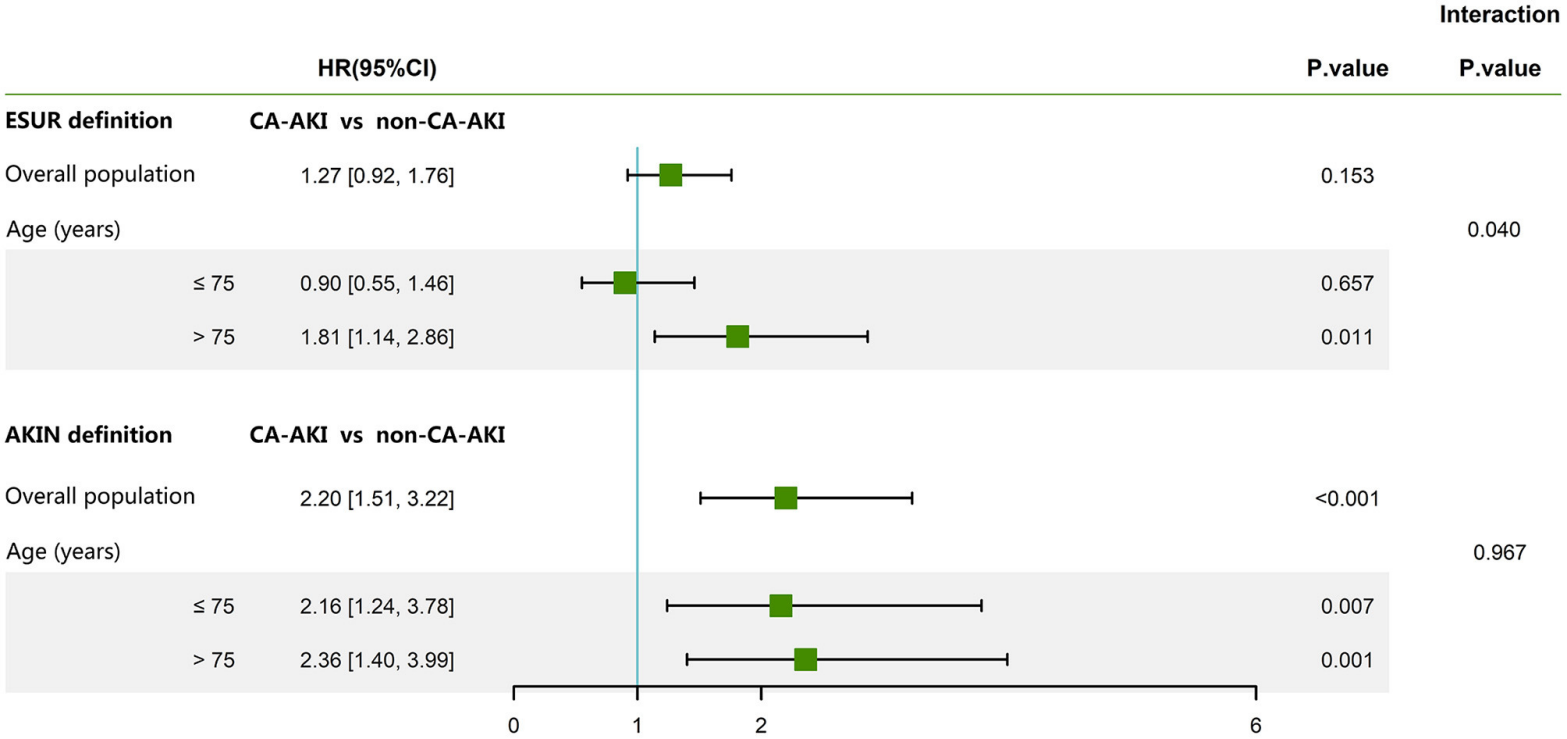
The association between the two classical definitions of CA-AKI and long-term mortality. The following parameters were adjusted: age >75 years, SCr >1.5 mg/dl, hemoglobin, multivessel disease, acute myocardial infarction, diabetes, and previous myocardial infarction. CA-AKI, contrast-associated acute kidney injury; SCr, serum creatinine; AKIN, Acute Kidney Injury Network; ESUR, European Society of Urogenital Radiology; HR, hazard ratio; CI, confidence interval.

After a median follow-up period of 2.1 years, 199 (3.9%) patients had died. After adjusting for age >75 years, previous MI, diabetes, SCr >1.5 mg/dl, hemoglobin, multivessel disease, and acute MI, CA-AKI as diagnosed by the AKIN definition (HR = 2.20; 95% CI: 1.51–3.22; *p* < 0.001) was identified as a significant risk factor for long-term mortality in the overall population. However, CA-AKI as diagnosed by the ESUR definition was not a significant risk factor after adjusting for these confounding factors (HR = 1.27; 95% CI: 0.92–1.76; *p* = 0.153) (Figure 3).

**Figure 4:**
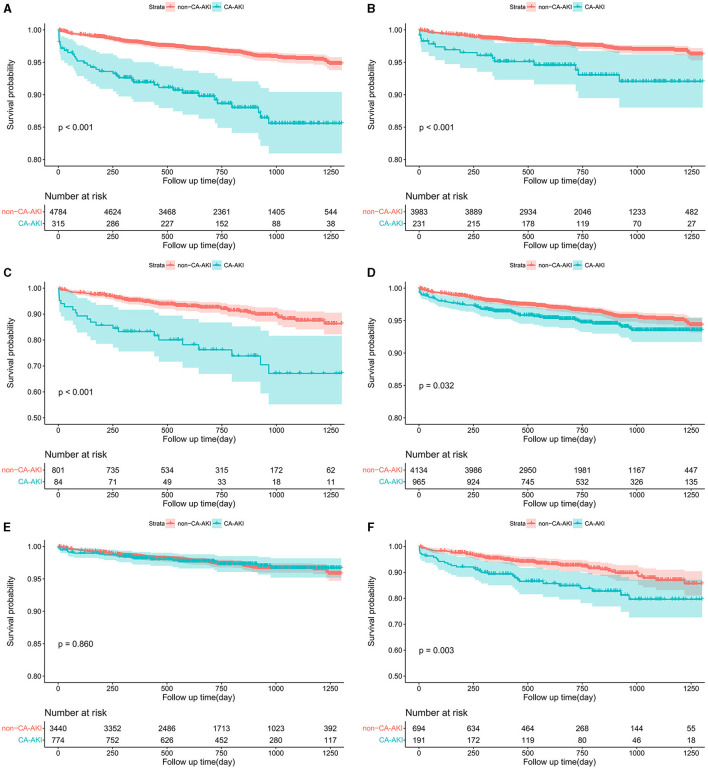
Kaplan–Meier curves for long-term mortality according to the AKIN definition in the overall population **(A)**, patients ≤75 years **(B)**, and patients >75 years **(C)** and according to the ESUR definition in the overall population **(D)**, patients ≤75 years **(E)**, and patients >75 years **(F)**. CA-AKI, contrast-associated acute kidney injury; AKIN, Acute Kidney Injury Network; ESUR, European Society of Urogenital Radiology.

Further interaction analysis of age revealed a significant interaction between age and ESUR definition for long-term mortality (*p* = 0.040) after adjusting for previous MI, diabetes, SCr >1.5 mg/dl, hemoglobin, multivessel disease, and acute MI. This association was not evident between age and the AKIN definition (*p* = 0.967) (Figure 3). When stratified by age, Cox regression analysis revealed that CA-AKI, as diagnosed by the AKIN definition, remained independently correlated with long-term mortality in patients >75 years of age (HR = 2.36; 95% CI: 1.40–3.99; *p* = 0.001) and ≤75 years of age (HR = 2.16; 95% CI: 1.24–3.78; *p* = 0.007). The ESUR definition was an effective predictor for long-term mortality in patients >75 years of age (HR = 1.81; 95% CI: 1.14–2.86; *p* = 0.011) but not for patients ≤75 years of age (HR = 0.90; 95% CI: 0.55–1.46; *p* = 0.657) (Figure 3). Figure 4 shows the Kaplan–Meier curves for long-term mortality according to both ESUR and AKIN definitions.

Next, we reclassified patients into three categories based on increasing SCr levels (group 1: an elevation of SCr <25% and <0.5 mg/dl; group 2: an elevation of SCr ≥25% but <0.5 mg/dl; group 3: an elevation of SCr ≥0.5 mg/dl). After adjusting for previous MI, diabetes, SCr >1.5 mg/dl, hemoglobin, multivessel disease, and acute MI, patients in group 2 showed a significantly higher risk of mortality than in group 1, but only in patients >75 years of age (HR = 1.80; 95% CI: 1.09–2.97; *p* = 0.023). In contrast, the risk of mortality was similar when compared between groups 1 and 2 for patients ≤75 years of age (HR = 0.60; 95% CI: 0.33–1.10; *p* = 0.101) (Supplementary Figure 2). The Kaplan–Meier curves showed the same results (Supplementary Figure 3).

## Discussion

This is the first study to demonstrate that the two classical definitions of CA-AKI have different prognostic impacts on long-term mortality when compared between elderly and non-elderly patients who were treated with elective PCI. Our data showed that when applying the AKIN definition, CA-AKI was independently correlated with long-term mortality not only in elderly patients (>75 years of age) but also in non-elderly patients (≤75 years of age). However, this association was only seen in elderly patients (>75 years of age) when the ESUR definition was applied.

CA-AKI is a well-established complication after PCI and is strongly linked with poor outcomes (1–5). However, in the absence of a standardized definition, the prevalence and prognostic value of CA-AKI exhibit significant variations (6–12). Among the various definitions of CA-AKI, the most commonly used definitions for patients who receive PCI are the two classical definitions proposed by the ESUR and AKIN (17, 18); these are both based on an absolute or relative increase in SCr. Numerous studies have demonstrated that the ESUR and AKIN definitions exhibit good prognostic values for predicting a poor prognosis (10, 21–26). However, some researchers have compared these two definitions and arrived at different conclusions. For example, Centola et al. investigated 402 ST-segment elevation MI (STEMI) patients who underwent primary PCI and detected a remarkable correlation between mortality and CA-AKI when applying the ESUR and AKIN definitions during a median 1-year follow-up period (27); in addition, the AKIN classification achieved better prognostic accuracy than the ESUR criteria (area under the receiver operating curve; 0.7984 vs. 0.7759; *p* = 0.0331). Similarly, Lun et al. showed that both the ESUR and AKIN definitions were associated with long-term mortality (28). However, it is noteworthy that the population attributable risk (PAR) for the AKIN definition was greater, thus indicating that this definition exhibited higher clinical relevance than the ESUR definition. Another prospective observational study, performed by Silvain et al., analyzed 1,114 patients who had been diagnosed with STEMI and arrived at a different conclusion in that the AKIN definition, but not the ESUR definition, could identify CA-AKI patients with a high in-hospital and 1-year mortality risk (29). In another study, Guillon et al. reported that CA-AKI was clearly associated with all-cause 6-month mortality when the AKIN definition was used (30). However, when applying the ESUR definition, only patients who showed an elevation of SCr ≥0.5 mg/dl had a significantly higher risk of mortality than those who showed an elevation of SCr ≥25% but <0.5 mg/dl (HR = 3.1; 95% CI: 1.5–6.6; *p* = 0.002) after diagnostic angiography or PCI. The mortality rate in the second group was found to be similar to that in patients without CA-AKI (30). In our present study, after adjusting for other potential confounders, we found that CA-AKI remained an independent risk factor for long-term mortality according to the AKIN definition in the overall population, but not for the ESUR definition.

In our present study, we found that the ESUR definition of CA-AKI was more lenient and associated with a significantly higher incidence than the AKIN definition (Figure 2). The prognostic impact of CA-AKI clearly depends on the definition used. The leniency of the ESUR definition led to greater sensitivity and identified a higher prevalence of CA-AKI but with less clinical relevance, thus increasing economic burden without impacting the hard clinical endpoints. Conversely, the more stringent AKIN definition was more specific and therefore resulted in a remarkable correlation with hard events but also a reduction in sensitivity; consequently, this definition may miss a substantial proportion of high-risk patients. Because of the different susceptibilities and prognoses of CA-AKI among individuals, the potential applicability of the CA-AKI definition might differ from one population to another. For example, Abe et al. found that CA-AKI was only a predictor for mortality in patients who presented with renal insufficiency (20); this relationship was not identified in patients with normal renal function. In the current study, the ESUR definition did not correlate with long-term mortality in the overall population. Furthermore, when interaction and stratified analysis of age were performed, we discovered the ESUR definition also had an important prognostic value for predicting long-term mortality in patients >75 years of age; no such relationship was observed in patients ≤75 years of age.

Next, we considered how the applicability of CA-AKI definitions varies in different populations. It is evident that the impact of the CA-AKI definition on morbidity and prognosis is highly dependent on the underlying renal function and renal functional reserve. Non-elderly patients often have a better underlying renal function and lower baseline SCr level; a 25% relative elevation in SCr might be equivalent to a minor increment in the absolute value of SCr, which could easily be reached but may be negligible and clinically insignificant. Therefore, some patients might be falsely diagnosed with CA-AKI (false positive) in such cases because of the low discriminating power based on relative increases in SCr. Consequently, many patients with a risk level comparable with that of individuals without CA-AKI were involved, thus reducing the predictive value of CA-AKI for adverse outcomes. In the present study, we found that non-elderly patients had significantly lower baseline SCr levels than elderly patients (Supplementary Figure 1). Many existing studies also support our hypothesis. For example, Budano et al. carried out a prospective observational study in 755 consecutive patients undertaking emergency or elective coronary angiography and identified that an absolute elevation of SCr ≥0.5 mg/dl was more relevant to in-hospital (11.5 vs. 5.0%) and follow-up mortality (19.6 vs. 8.1%) than a relative elevation of ≥25% in SCr (31). Furthermore, patients who met a ≥25% but not 0.5 mg/dl increment in SCr showed comparable levels of cardiovascular mortality as those without CA-AKI (4.3 vs. 4.1%). A similar conclusion was reported in another prospective study that involved patients undergoing PCI in that both a >25% or >0.5 mg/dl increment in SCr was correlated with 6-month major adverse cardiovascular events and all-cause mortality (32). Furthermore, when individuals were reclassified into three prognostic categories, these researchers found that the prognoses of patients who reached a relative increase in SCr >25% but not an absolute SCr increase >0.5 mg/dl were similar to those without CA-AKI. Moreover, the clinical relevance of the subgroup with an increment of SCr ≥25% but <0.5 mg/dl was profoundly challenged in a large collaborative registry by Slocum et al. (9). In addition, non-elderly patients commonly had a better renal functional reserve and recovered better from CA-AKI than elderly patients (33), thus improving the prognosis of patients with CA-AKI (34–36). A meta-analysis of 17 studies involving patients who had been diagnosed with CA-AKI also reported that the risk of failure to restore renal function in the elderly was greater than that in non-elderly patients (pooled relative risk: 1.28; 95% CI: 1.06–1.55; *p* < 0.05) (37).

Our results also indicate that patients with an elevation of SCr ≥25% but <0.5 mg/dl did not exhibit clear differences in long-term mortality when compared with those without CA-AKI in patients ≤75 years of age. However, in patients >75 years of age, we observed distinct prognoses in the two subgroups. With regard to the AKIN definition, we found that CA-AKI was significantly correlated with long-term mortality in patients >75 years of age and in patients ≤75 years of age. Therefore, we recommend using the AKIN definition for the prediction of long-term mortality in the non-elderly population to avoid the inclusion of patients with “average risk.” Furthermore, using relatively stringent criteria in the elderly population, such as the AKIN definition, may lead to a missed diagnosis and a delay in prophylactic measures among high-risk patients. However, we found that more lenient criteria provided by the ESUR could sensitively identify high-risk elderly patients with long-term mortality. Consequently, in our opinion, the ESUR definition may represent a better alternative for this patient group rather than the AKIN definition.

There are several limitations to this study that need to be considered. First, the complete AKIN definition involves SCr and urinary output; the latter parameter was not included in our present study. However, it must be noted that urinary output is affected to a large extent by hydration status and diuretics. Previous research demonstrated a poor correlation between CA-AKI according to urinary output criteria alone as part of the AKIN definition and mortality (38, 39). Second, despite the fact that we adjusted data for potential confounders, other residual confounding factors may still exist and may have affected our results. Thus, the findings of the present study should be considered from the viewpoint of hypothesis generation. Third, we performed *post-hoc* power analysis of our data and found that our analyses were underpowered (51.5%) with regard to detecting an association between CA-AKI (according to the ESUR definition) and long-term mortality, as compared with the normal standard of 80%. This was probably due to the low long-term mortality (3.9%) observed in our study population. Adequate power (84.3%) could be obtained when the long-term mortality rate increased to 8.6%. Therefore, our non-significant finding might be attributed to insufficient statistical power. Considering this limitation, the findings of our study are important but should be interpreted with caution. Further studies now need to investigate the specific association between CA-AKI (according to the ESUR definition) and long-term mortality. Fourth, this study was carried out in single center; our results therefore need to be validated in a well-designed prospective featuring a large cohort and multiple centers. Finally, our conclusions may only apply to patients undergoing elective PCI. The prognostic impact of CA-AKI (as diagnosed by different definitions) on long-term mortality in other populations requires further investigation. Despite these limitations, our findings provided important insights into the applicability of the CA-AKI definition in the elderly and non-elderly populations.

## Conclusion

Our study is the first to report that the AKIN definition, as a stringent definition of CA-AKI, was significantly associated with long-term mortality in both non-elderly and elderly patients. However, in elderly patients, the more lenient definition provided by the ESUR was also significantly correlated with long-term mortality, which could sensitively identify high-risk elderly patients and may provide a better alternative.

## Data Availability Statement

The raw data supporting the conclusions of this article will be made available by the authors, without undue reservation.

## Ethics Statement

The studies involving human participants were reviewed and approved by Fujian Provincial Hospital Ethics Committee. The patients/participants provided their written informed consent to participate in this study.

## Author Contributions

YG and KL designed and conducted this study. HH and ZY performed the statistical analysis. XL, CH, SZ, and MLu helped to collect the data. MLi and LZ were involved in data cleaning and follow-up. YG, KL, and ZY revised the manuscript. All authors contributed to the article and approved the submitted version.

## Funding

This study was supported by the National Natural Science Foundation of China General Program (grant numbers: 81873495 and 82070375), the Heart Failure Center Research Fund of Fujian Provincial Hospital (supported by Fujian Provincial Department of Finance), the Natural Science Foundation of Fujian Province (2018J01242), the high-level hospital foster grants from Fujian Provincial Hospital, Fujian province, China (grant number: 2020HSJJ05), and the Fujian provincial health technology project (grant number: 2019-ZQN-10).

## Conflict of Interest

The authors declare that the research was conducted in the absence of any commercial or financial relationships that could be construed as a potential conflict of interest.

## Publisher's Note

All claims expressed in this article are solely those of the authors and do not necessarily represent those of their affiliated organizations, or those of the publisher, the editors and the reviewers. Any product that may be evaluated in this article, or claim that may be made by its manufacturer, is not guaranteed or endorsed by the publisher.
