# Supplementary material for: A Comparison Between Two Definitions of Contrast-Associated Acute Kidney Injury for Long-Term Mortality in Elderly and Non-elderly Patients After Elective Percutaneous Coronary Intervention
Source: Front Cardiovasc Med. 2021 Sep 27;8:720857. doi: 10.3389/fcvm.2021.720857 (PMC8504683; doi:10.3389/fcvm.2021.720857)
Supplement: Supplementary file 1 [file Data_Sheet_1.pdf]

Supplementary Material

Supplementary Figures

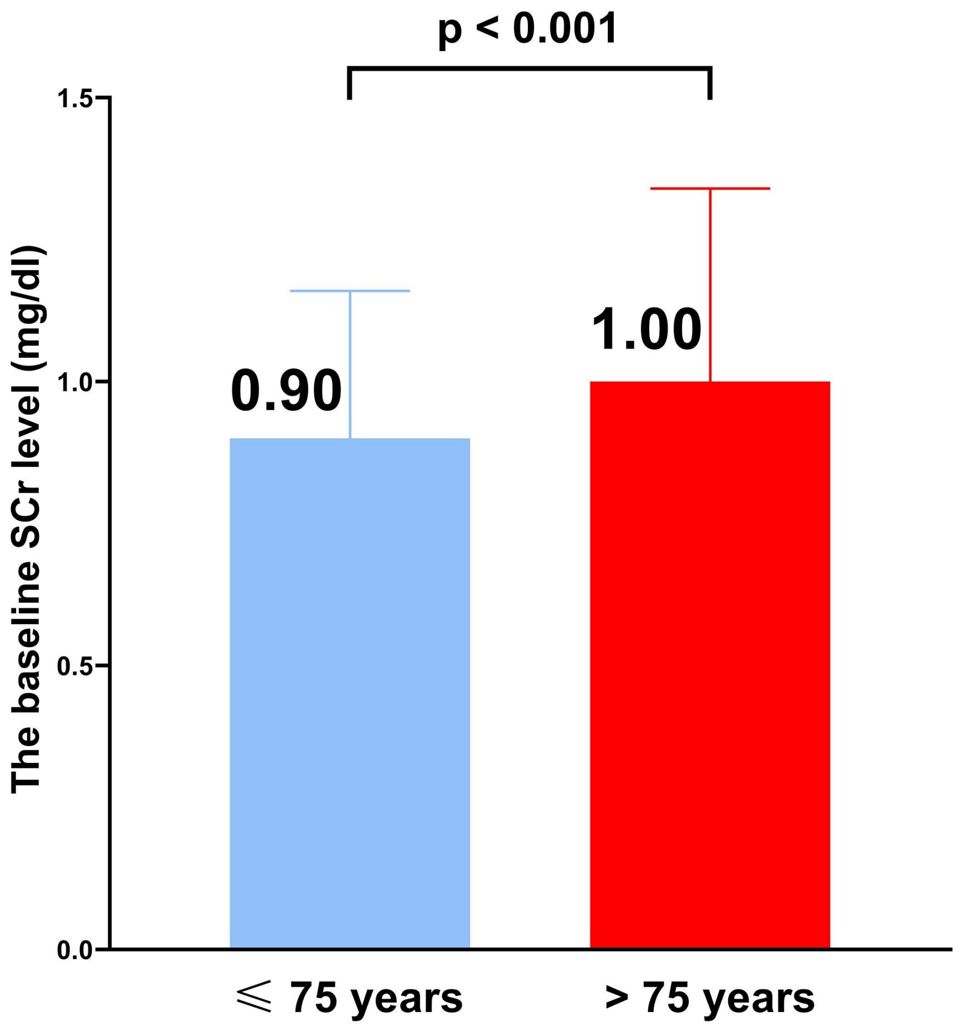

Supplementary Figure 1. Comparison of baseline serum creatinine level between patients ≤75 years-of-age and >75 years-of-age. SCr, serum creatinine.

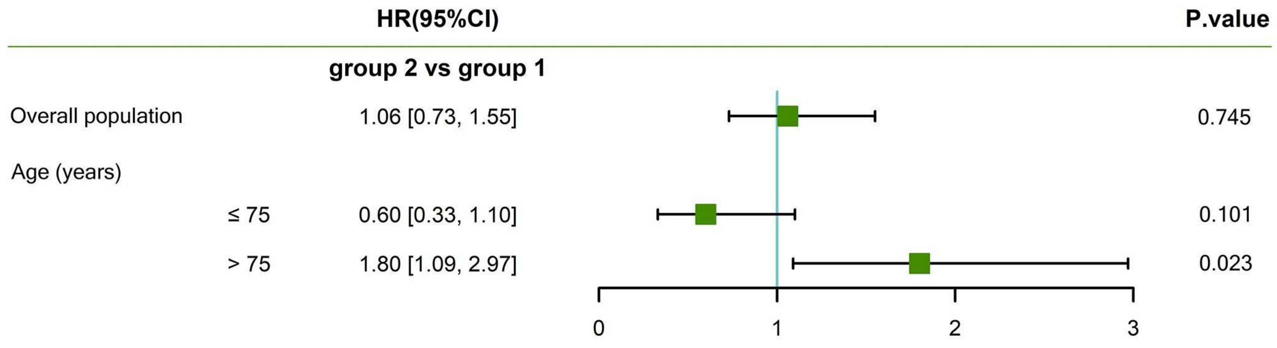

Supplementary Figure 2. The association between reclassified groups based on increased SCr levels and long-term mortality in patients ≤75 years-of-age or patients >75 years-of-age. The following parameters were adjusted: previous myocardial infarction, diabetes, SCr >1.5mg/dl, hemoglobin, multivessel disease, and acute myocardial infarction. Group 1: an elevation of SCr <25% and <0.5 mg/dl; Group 2: an elevation of SCr ⩾25% but <0.5 mg/dl. CA-AKI, contrast-associated acute kidney injury; ESUR, European Society of Urogenital Radiology; SCr, serum creatinine; HR, hazard ratio; CI, confidence interval.

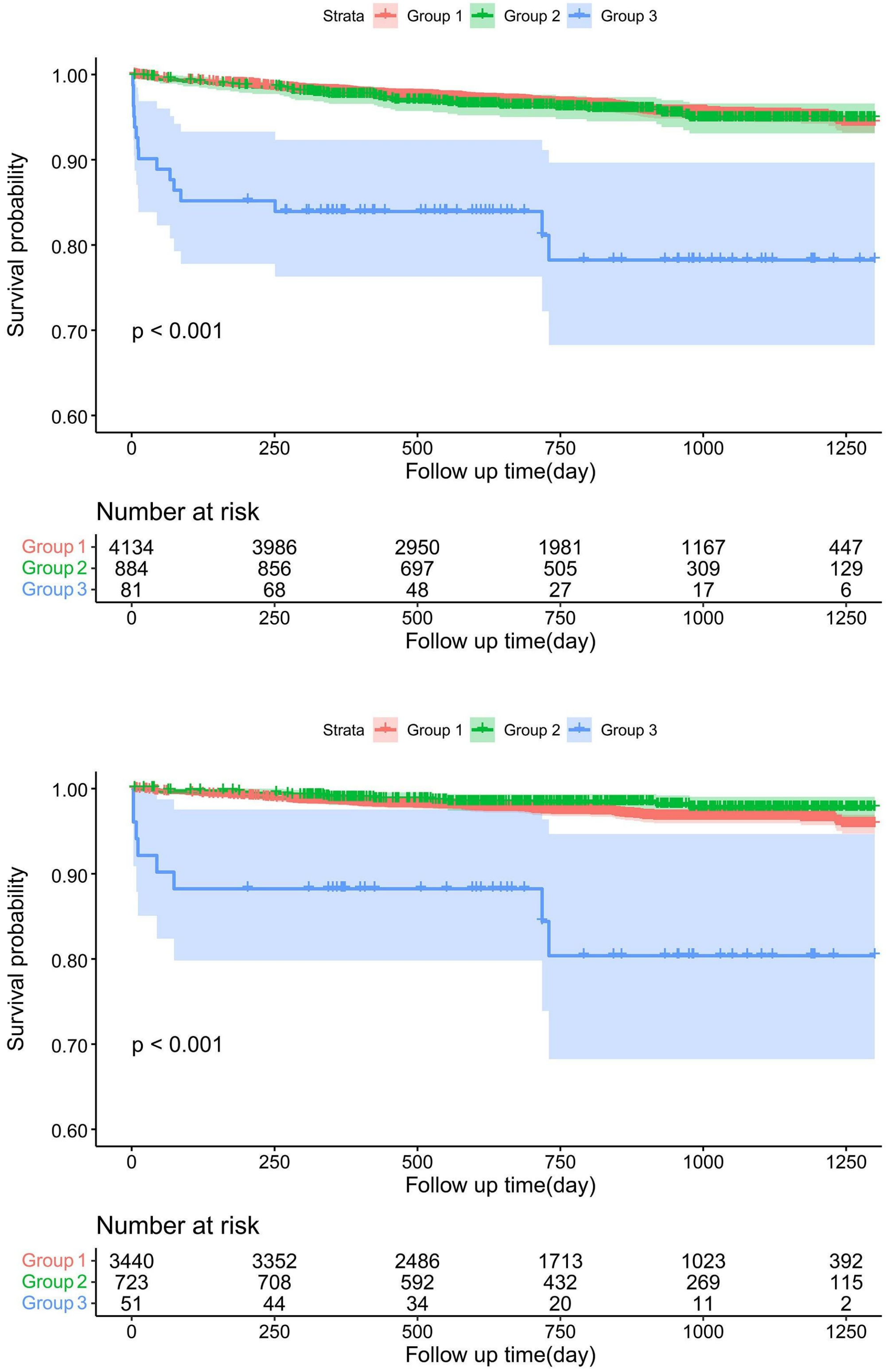

Supplementary Figure 3. Kaplan-Meier curves according to reclassified groups based on increased SCr levels. **(A)** The overall population. **(B)** Patients ≤75 years-of-age. **(C)** Patients >75 years-of-age. Group 1: an elevation of SCr <25% and <0.5 mg/dl; Group 2: an elevation of SCr ≥25% but <0.5 mg/dl; Group 3: an elevation of SCr ≥0.5 mg/dl. SCr, serum creatinine. **(Continued on next page)**

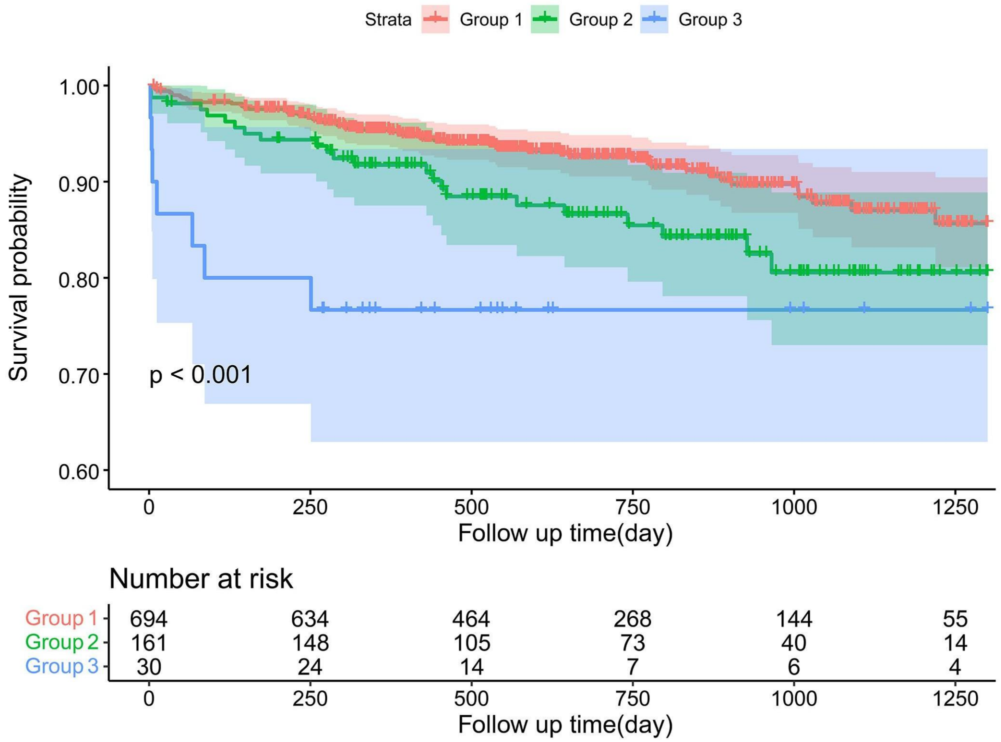

Supplementary Figure 3. (Continued) Kaplan-Meier curves according to reclassified groups based on increased SCr levels. **(A)** The overall population. **(B)** Patients ≤75 years-of-age. **(C)** Patients >75 years-of-age. Group 1: an elevation of SCr <25% and <0.5 mg/dl; Group 2: an elevation of SCr ⩾25% but <0.5 mg/dl; Group 3: an elevation of SCr ≥0.5 mg/dl. SCr, serum creatinine.
